# Exploring cognitive integration of basic science and its effect on diagnostic reasoning in novices

**DOI:** 10.1007/s40037-016-0268-2

**Published:** 2016-05-31

**Authors:** Kristina Lisk, Anne M. R. Agur, Nicole N. Woods

**Affiliations:** Rehabilitation Sciences Institute, Faculty of Medicine, University of Toronto, Toronto, Ontario Canada; Department of Surgery, Faculty of Medicine, University of Toronto, Toronto, Ontario Canada; Department of Family and Community Medicine, Faculty of Medicine, University of Toronto, Toronto, Ontario Canada; The Wilson Centre for Health Professions Education, Toronto General Hospital, Toronto, Ontario Canada

**Keywords:** Basic sciences, Diagnostic reasoning, Diagnostic justification, Clinical reasoning, Integration

## Abstract

Integration of basic and clinical science knowledge is increasingly being recognized as important for practice in the health professions. The concept of ‘cognitive integration’ places emphasis on the value of basic science in providing critical connections to clinical signs and symptoms while accounting for the fact that clinicians may not spontaneously articulate their use of basic science knowledge in clinical reasoning. In this study we used a diagnostic justification test to explore the impact of integrated basic science instruction on novices’ diagnostic reasoning process. Participants were allocated to an integrated basic science or clinical science training group. The integrated basic science group was taught the clinical features along with the underlying causal mechanisms of four musculoskeletal pathologies while the clinical science group was taught only the clinical features. Participants completed a diagnostic accuracy test immediately after initial learning, and one week later a diagnostic accuracy and justification test. The results showed that novices who learned the integrated causal mechanisms had superior diagnostic accuracy and better understanding of the relative importance of key clinical features. These findings further our understanding of cognitive integration by providing evidence of the specific changes in clinical reasoning when basic and clinical sciences are integrated during learning.

## Essentials

- Cognitive integration of basic and clinical sciences supports diagnostic reasoning in novices.
- Novices understand the relative importance of key clinical features for disease categories when instruction supports cognitive integration of basic and clinical sciences.
- A simple diagnostic justification test can be used to indirectly capture novices’ integrated basic science knowledge in clinical diagnosis.

## Introduction

Integration of basic science is increasingly being recognized as important for practice in the health professions [1]. As such, better integration of basic science disciplines with clinical content has become a central characteristic of curriculum reform [2]. Common integration strategies include problem-based learning, early exposure to real and simulated clinical experiences, rearrangement of basic science and clinical curricula, and shared teaching [3, 4]. While there is limited empirical evidence for the value of many of these curricular integration efforts, learners have been found to benefit when learning is based on the concept of ‘cognitive integration’. In contrast to the more common horizontal or vertical integration, ‘cognitive integration’ captures the understanding that the integration of basic and clinical sciences is a cognitive activity that occurs within the learner, not in the curriculum [3]. Cognitive integration is supported through day-to-day micro-level teaching and involves specific pedagogical strategies that purposefully link the basic and clinical sciences [3].

There is considerable laboratory evidence that cognitive integration supports diagnostic reasoning in novices [5–10]. For example, when compared with students who learned only clinical signs and symptoms, students who learned neurological and rheumatological diseases through integrated basic science descriptions had superior diagnostic accuracy 1‑week after learning. By understanding the causal mechanisms that govern why clinical features are associated with a specific disease, students’ diagnostic decisions can be based on what ‘makes sense’ rather than on the memorization of isolated features [7, 8]. The purposeful and explicit integration of basic and clinical sciences during teaching essentially allows the learner to develop a coherent mental representation of the disease category [6, 8].

Experimental findings demonstrating the value of this conceptual coherence have been consistent with undergraduate populations from a number of areas of medicine and other health disciplines, including dentistry, neurology, and rheumatology [6, 9]. The results of these studies suggest that immediately after learning novices may rely on an analytical feature counting strategy to arrive at a diagnosis, but after the passage of time their reasoning strategy shifts to rely on a more holistic understanding of disease categories in order to maintain diagnostic performance [6]. While this model of reasoning is supported in the pattern of performance across studies, it has been difficult to provide an explicit measure of how students use their coherent mental representation to arrive at the correct diagnosis. This is because the model of conceptual coherence does not necessitate the overt application of basic science knowledge in diagnosis. Rather, the model suggests that novice diagnosticians use basic science knowledge unconsciously and automatically to reorganize and reconstruct diagnostic feature lists associated with abnormal functioning when solving clinical problems [7]. This cognitive process is evident by learners’ ability to arrive at a correct diagnosis but might not be easily expressed. Thus, directly asking learners how or if they used their basic science knowledge to solve a case might not lead to greater insights regarding cognitive integration or conceptual coherence.

An explanation consistent with conceptual coherence is that, while impactful, basic science knowledge is less likely to be articulated in a think aloud protocol unless the cases are particularly complex. Schmidt et al. [11] theorized that experts’ basic science knowledge becomes encapsulated under clinical concepts as a result of repeated clinical exposure. However, when experts are faced with a challenging clinical problem they revert to their basic science knowledge for an explanation. This is supported in studies that have compared think aloud protocols from novices and experts as they reason through difficult clinical problems [12, 17]. For example, a study that compared reasoning strategies of junior residents to experienced clinicians as they worked through complex nephrology problems found that increased experience was associated with superior diagnostic performance and more extensive use of causal explanations [12].

Recently, Williams and Klamen [13] have described a written diagnostic justification task, intended to make students’ diagnostic strategy explicit. In this task, students were asked to identify their diagnostic strategy by explaining how they used patient and laboratory data to move from initial differential diagnoses to a final diagnostic decision. It was found that students’ diagnostic justification scores were highly correlated to the final comprehensive exam score [13]. Moreover, the relative contribution of biomedical knowledge and clinical cognition on students’ diagnostic strategy has been investigated using structural equation modelling. This structural equation modelling study revealed a small correlation between biomedical and clinical knowledge in the first two years of training, but found that both constructs demonstrated a moderate relationship with diagnostic justification ability of fourth year students [14]. These findings suggest that the diagnostic justification task appears to capture the use of basic science knowledge in clinical diagnosis. Based on these findings, it plausible that a diagnostic justification task could provide a way to explicitly capture the impact of integrated basic science knowledge on novices’ diagnostic reasoning process.

In the present study, we aimed to extend previous work on cognitive integration using new learning materials teaching musculoskeletal pathologies with allied health students. In addition, we aimed to further our understanding of cognitive integration and conceptual coherence by using a diagnostic justification task to investigate the impact of integrated basic science instruction on novices’ diagnostic reasoning process. We hypothesized that students who are taught musculoskeletal conditions using basic science descriptions would have superior diagnostic accuracy after a time delay compared with those who are only taught the clinical features. It was expected that this effect would be present even though learners’ memory of clinical features associated with each musculoskeletal condition may decline over time. Furthermore, we anticipated that the diagnostic justification task would allow for explicit measurement of the impact cognitive integration has on novices’ diagnostic reasoning process, allowing for the possibility that students would use their basic science knowledge, without necessitating its articulation.

## Methods

### Participants

Forty-five first and second year massage therapy students from Humber College, Toronto participated in this study. All students had completed the same introductory musculoskeletal anatomy course. The students were assumed to have a basic understanding of the bones, joints, and muscles of the hand but had minimal, if any, prior experience with the musculoskeletal pathologies selected for the learning materials. Students received a $ 30 Campus Bookstore gift card for participating. Human research ethics approval was obtained from Humber College and participation was completely voluntary.

### Learning materials

Two learning conditions were created for the purpose of this study; an integrated basic science (BaSci) condition and a clinical science only (CS) condition. Participants in both learning conditions were taught the clinical features of four confusable musculoskeletal pathologies: Dupuytren’s contracture, carpal tunnel syndrome, Guyon’s canal syndrome, and pronator teres syndrome. The learning material for each of the pathologies in the BaSci group included an integrated review of relevant anatomical structures, clinical features, and the underlying causal mechanisms (anatomical pathology) of each feature. The learning material for the CS group used the same descriptions and images/video clips for the clinical features of each pathology; however, the anatomy and underlying causal mechanisms were excluded. To equalize the learning time between the two conditions, the CS group was taught epidemiology and potential treatment options for each of the four pathologies. In both learning conditions participants were not told explicitly which clinical features were key to making a correct diagnosis. An example of learning material for both groups is shown in Tab. 1. The learning materials consisted of images and video clips accompanied by audio recordings (19 minutes in length) that narrated the written material on each slide. Participants were given an unlimited amount of time to study each slide but were not permitted to click backwards through the learning materials. This was done in an effort to control the time on task between the groups. Two textbooks, the Anatomical Basis of Neurologic Diagnosis [15] and Clinically Oriented Anatomy [16] were content references for the learning materials. Both learning conditions were reviewed for clarity and accuracy by an experienced physical medicine and rehabilitation clinician and a clinical anatomist.

**Tab. 1:** Sample explanations for Dupuytren contracture explained in the two learning conditions

**Integrated Basic Science Group**
Dupuytren contracture presents as painless nodular thickenings of the palmar aponeurosis that adheres to the skin. No pain is associated with the disease since the nerves of the hand which transmit pain information to the brain are not affected. Gradually, thickening and progressive shortening (contracture) of the longitudinal bands produces raised ridges in the palm of the hand. Fibrosis degeneration and shortening of the longitudinal bands causes partial flexion of the affected fingers at the metacarpophalangeal and proximal interphalangeal joints. With progressive disease, a flexion deformity will develop and as a result the patient will report an inability to fully extend the affected fingers at the metacarpophalangeal and proximal interphalangeal joints. The flexion deformity is caused by the shortening of the longitudinal bands of the palmar aponeurosis. The flexion deformity limits the person’s ability to fully open their hand, making it difficult to grasp large objects. In Dupuytren contracture there are no sensory changes observed in the hand. This is because the contracture does not affect the nerves of the hand that are responsible for supplying sensory information to the skin
**Clinical Science Only Group**
Dupuytren contracture presents as painless nodular thickenings that adhere to the skin. Gradually, patients present with raised ridges in the palmar skin that extend from the proximal part of the hand to the base of the fingers. In patients’ affected fingers, partial flexion occurs at the metacarpophalangeal and proximal interphalangeal joints. With progressive disease, a flexion deformity can develop and patients will report an inability to fully extend the affected fingers at the metacarpophalangeal and proximal interphalangeal joints. The disease can occur in both hands but is generally not symmetric in severity. The ring finger is most commonly involved followed by the little finger. Patients typically have a difficult time grasping large objects. There are no sensory changes observed in this disease

### Testing materials

Three tests were used in this study.

1. Diagnostic accuracy test: To test diagnostic accuracy, participants were presented with 15 clinical cases and were asked to choose the correct diagnosis from a list of four pathologies. Each case description included the age, sex, a minimum of three clinical features, and an image or video clip of the patient’s hand presentation. For counterbalancing purposes, two versions (A and B) were created and matched for difficulty. Both tests were reviewed for accuracy by a clinical anatomist and piloted by 22 undergraduate students. Analysis of the pilot data revealed no difference between test A and B.
2. Memory test: To measure participants’ recall of clinical features they were asked to choose the correct features for each of the pathologies from a list of 16 features. The same list was provided for all four musculoskeletal pathologies.
3. Diagnostic justification test: This test aimed to explicitly capture the participants’ diagnostic reasoning process when explaining a correct diagnosis. Participants were provided with an image of a patient’s hand presentation and were told the correct diagnosis. Participants were then asked to provide the patient with a written explanation of their diagnosis, being as specific as possible. These explanations were typed into a text box located below the image of the patient’s hand presentation. All four pathologies were tested in the same manner with participants having no time or word count restrictions to provide their response. This simplified diagnostic justification measure was specifically developed for this study and was considered to be appropriate for this context. The prompt used for each question on this test was deliberately left vague in an effort not to influence participants’ responses and to avoid intentional learning instructions [17]. Further, unlike the diagnostic justification task used by Williams & Klamen [13], we did not require students to provide a diagnosis or a differential diagnosis nor were students prompted to list key clinical findings (positive or negative).

The learning and testing materials were presented using a customized software programme which enabled us to control the minimum amount of learning time for each participant, record reaction times, and track participant responses.

### Protocol

Upon consent, participants were randomly allocated 1:1 into the BaSci or the CS group. This study was completed in cohorts up to six participants at a time. Each participant was seated at an individual table and was provided with a laptop computer, headphones, and instructions for viewing and testing. Before starting the learning materials participants completed a prior knowledge test and a basic hand anatomy tutorial and quiz. The prior knowledge test consisted of five clinical cases and used the same format as described for the diagnostic accuracy test. The basic hand anatomy tutorial and quiz were created to review anatomical terminology and the bones, joints, and joint movements of the hand. At the end of the tutorial participants completed seven multiple-choice questions on basic hand anatomy. The computer programme scored the quiz and required participants to achieve a minimum of 86 % (6 out of 7) in order to proceed to the learning phase of the study. Participants who did not achieve 86 % on their first attempt were redirected to the beginning of the tutorial and were instructed to review the material. Following the second attempt on the quiz all participants were directed to the learning phase. Immediately after the learning phase, participants completed the diagnostic accuracy test (test A or B) followed by the memory test. One week later, participants returned to complete the diagnostic accuracy test (test A or B), followed by the diagnostic justification test, and the memory test. Participants who had taken diagnostic accuracy test A the previous week were given test B, and vice versa. On both immediate and delayed testing, all test items were presented one at a time, in random order, and no time restrictions were imposed.

### Analysis

**Fig. 1:**
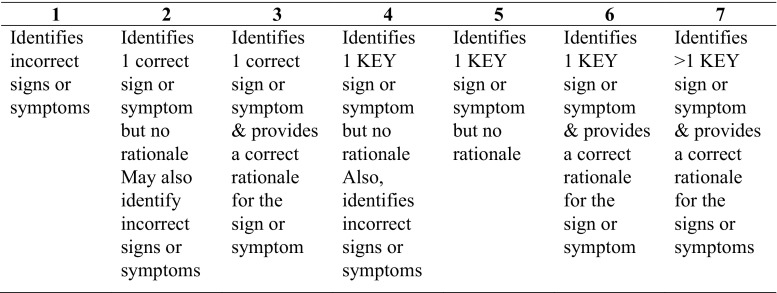
Likert scale used to score the diagnostic justification test

An independent samples t‑test was used to compare the prior knowledge test scores of the BaSci and CS group. For each participant, the number of correct responses on the diagnostic accuracy and memory tests was calculated. The results on these two tests were analyzed separately using a 2 × 2 repeated measures ANOVA, with the learning group (BaSci and CS) as the between-subject variable and time (immediate vs. delayed) as the within-subject variable. A series of planned t‑tests were also performed. The same analysis was used to compare the amount of time it took participants to complete the diagnostic test on immediate and delayed testing. Based on pilot data, a seven-point Likert scale was created by the research team to score participants’ diagnostic justification responses (Fig. 1). The scale ranged from one (identifies incorrect sign/symptoms) to seven (identifies more than one key sign/symptoms for the pathology and provides a correct rationale for each sign/symptom). Two independent, blinded raters used the scale to score all responses. To assist with grading, raters were provided with a list of clinical features associated with each of the pathologies with the key clinical features highlighted. Intra-class correlation was calculated to measure agreement between the raters. The average of the raters’ scores for each participant was subject to an independent samples t‑test to compare the type of information participants used to justify their diagnosis. Pearson’s correlations were calculated for both learning groups to measure the relationship between participants’ diagnostic accuracy and diagnostic justification scores and diagnostic accuracy and time to complete the diagnostic tests.

## Results

A *priori* it was decided that participants would be excluded from the final analysis if they did not complete testing at both time points or if they were identified as an outlier on either the diagnostic or recall test. One participant did not return to complete follow-up testing and a box plot analysis identified one participant as an outlier on the first recall test. A total of 43 participants were included in the final analysis.

The BaSci group (*n* = 22) scored 48 % and the CS group (*n* = 21) 39 % on the prior knowledge test. A comparison of these scores revealed no difference (*p* = 0.12).

On the diagnostic accuracy test, participants in the BaSci group more accurately diagnosed the musculoskeletal pathologies on both immediate and delayed testing compared with the CS group (Tab. 2). The ANOVA showed a significant main effect of time,* F*_1, 42_ = 12.3, *p* = 0.001, $\eta _{\mathrm{p}}^{2}$= 0.23 and learning group, *F*_1, 42_ = 11.2, *p* = 0.002, $\eta _{\mathrm{p}}^{2}$= 0.21. The effect size of the difference for the learning groups was in the large effect range (d = 0.82).

**Tab. 2:** Average scores on the diagnostic accuracy, memory, and diagnostic justification tests

		Immediate		Delayed	
		Mean	SD	Mean	SD
Diagnostic accuracy	BaSci group (*n* = 22)	0.73	0.15	0.65	0.24
	CS group (*n* = 21)	0.58	0.19	0.46	0.14
Memory	BaSci group (*n* = 22)	0.66	0.17	0.58	0.17
	CS group (*n* = 21)	0.47	0.11	0.51	0.11
Diagnostic justification	BaSci group (*n* = 22)	–	–	3.9	1.04
	CS group (*n* = 21)	–	–	3.1	0.96

Time taken to complete the diagnostic accuracy test immediately after learning and one week later differed between the BaSci (8.5/8.1 min) and CS (7.6/6.4) groups. The ANOVA showed a significant main effect of group,* F*_1, 42_ = 4.8, *p* = 0.03, but there was no significant correlation between diagnostic performance and time to complete the diagnostic tests.

On the memory test, the BaSci group outperformed the CS group (Tab. 2). The ANOVA showed a significant main effect of learning group,* F*_1, 42_ = 12.4, *p* = 0.001, $\eta _{\mathrm{p}}^{2}$= 0.23, and a significant interaction between time and learning group,* F*_1, 42_ = 6.3, *p* = 0.02, $\eta _{\mathrm{p}}^{2}$= 0.13. To determine what was driving the interaction, a series of planned t‑tests were performed. An independent samples t‑test revealed the BaSci group did significantly better than the CS group on immediate testing only (*p* < 0.01).

As shown in Tab. 2, the BaSci group also outperformed the CS group on the diagnostic justification test (*p* = 0.01). The effect size of the difference for the learning groups was in the moderate to large effect range (0.74). Explanations provided by the BaSci group included one key feature for each disease category along with an incorrect feature(s). In contrast, the explanations by the CS group included the identification of one correct feature; however, the feature was common to more than one disease category. Agreement between the two independent raters was high (ICC = 0.90). A significant correlation was found between students’ diagnostic justification and diagnostic accuracy scores one week after initial learning for both the BaSci (r = 0.70, *n* = 22, *p* < 0.001) and CS groups (r = 0.51, *n* = 21, *p* < 0.02). These data provide some validity evidence for the simplified diagnostic justification test used in this study.

## Discussion

The BaSci group outperformed the CS group on the diagnostic accuracy tests. One week after initial learning, both groups experienced a drop in performance; however, the smallest decline was observed in the BaSci group. Students who received integrated instruction also outperformed students who were only taught the clinical features of the pathologies on the basic memory test. However, this difference was no longer evident one week later. Thus, as predicted, students in the BaSci group were able to maintain superior diagnostic performance after a time delay, despite showing no advantage of remembering the clinical features for the pathologies learned. These results support the model of conceptual coherence and provide converging evidence for the value of basic science in clinical reasoning [7, 8].

Students who were taught using integrated basic science also outperformed those who were only taught the clinical features on the diagnostic justification test. Both groups identified correct features on the test, but those who received integrated instruction identified key diagnostic features rather than features that were common across disease categories. As hypothesized, the BaSci group was able to more accurately justify the pathologies learned without overtly using their basic science knowledge. These results provide insight on how learners’ integrated basic science knowledge is used to make more accurate clinical decisions. This finding also furthers our understanding of conceptual coherence by providing explicit evidence of specific changes that occur in clinical reasoning when instruction supports the integration of basic and clinical science knowledge.

Previous work in clinical reasoning has shown that better conceptual coherence results in novices exhibiting expert-like behaviour when solving clinical problems, including more automatic and holistic processing [7, 18]. The findings of the current study suggest this may be due to the learners’ greater understanding of the relative importance of key clinical features as evidenced by the students’ explanations on the diagnostic justification test. Furthermore, the strong correlation found between the BaSci group’s diagnostic accuracy and diagnostic justification scores are consistent with predictions made by a recent structural equation modelling study [14], thereby providing evidence that diagnostic justification can indirectly capture the use of learners’ integrated basic science knowledge in clinical diagnosis.

This study has limitations that should be noted. The simple diagnostic justification test used in this study was created specifically for this experiment and these learning materials. The results of the test cannot be taken as a generalizable measure of the participants’ justification abilities. We cannot conclude that integrated basic science instruction leads to better diagnostic justification in all settings or for all cases. Further, the diagnostic justification test was completed one week following initial instruction, immediately after the diagnostic accuracy test. Studies on non-analytical reasoning have demonstrated that novice problem solving is influenced to some degree by similarity to exemplars in memory [19]. Thus, it is possible that exposure to the clinical descriptions and pictures on the diagnostic accuracy test influenced students’ explanations on the justification task. Students were also incentivized to participate and it is unknown whether the same results would be observed in a general setting. In addition, all aspects of this study took place in an artificial learning environment and the learning materials were tightly controlled using customized software. These learning conditions and materials may not reflect how learning would occur in a classroom setting.

The importance of integrating basic science instruction with clinical training throughout undergraduate curricula is well recognized and several strategies that aim to integrate these two knowledge domains have been described [4, 20, 21]. However, curricular innovations that merely create proximity between the basic and clinical sciences have not been found to significantly improve learners’ integrated knowledge [22, 23]. In contrast, the current study shows that when basic and clinical science knowledge is cognitively integrated, learners develop better conceptual coherence and as a result have superior diagnostic abilities. Further, by teaching students the causal basic science mechanisms they understood the relative importance of key clinical features for disease categories and we suggest that they use this knowledge to make more accurate clinical decisions. This highlights the utility of integrated basic science knowledge and emphasizes the importance of purposefully linking basic and clinical science instruction in day-to-day teaching. Moreover, a simple diagnostic justification task has been identified as an additional measure that educators can use to assess learners’ grasp of integrated instructional materials without reliance on explicit articulation.

## Conclusion

This study demonstrates the positive impact of integrating basic anatomical education and clinical science instruction on students’ diagnostic reasoning ability in addition to diagnostic accuracy. The findings of this study further our understanding of conceptual coherence by providing explicit evidence of the advantage learners have when basic science knowledge is cognitively integrated. Future research should explore potential learning strategies that will promote the development of integrated basic science knowledge.

## References

[1] Association of American Medical Colleges and the Howard Hughes Medical Institute. Report of Scientific Foundations for Future Physicians Committee. Washington, DC: Association of American Medical Colleges; 2009 [cited 2015 August 29]. 43 p. Available from: http://www.hhmi.org/news/aamc-hhmi-committee-defines-scientific-competencies-future-physicians

[2] Anderson MB, Kanter SL (2010). A Snapshot of Medical Student Education in the United States and Canada: Reports From 128 Schools. Acad Med.

[3] Kulasegaram KM, Martimianakis MA, Mylopoulos M, Whitehead CR, Woods NN (2013). Cognition before curriculum: Rethinking the integration of basic science and clinical learning. Acad Med.

[4] Brauer DG, Ferguson KJ (2015). The integrated curriculum in medical education: AMEE guide no. 96. Med Teach.

[5] Woods NN, Brooks LR, Norman GR (2005). The value of basic science in clinical diagnosis: Creating coherence among signs and symptoms. Med Educ.

[6] Woods NN, Neville AJ, Levinson AJ, Howey EH, Oczkowski WJ, Norman GR (2006). The value of basic science in clinical diagnosis. Acad Med.

[7] Woods NN (2007). Science is fundamental: the role of biomedical knowledge in clinical reasoning. Med Educ.

[8] Baghdady MT, Pharoah MJ, Regehr G, Lam EW, Woods NN (2009). The role of basic sciences in diagnostic oral radiology. J Dent Educ.

[9] Baghdady MT, Carnahan H, Lam EW, Woods NN (2013). Integration of basic sciences and clinical sciences in oral radiology education for dental students. J Dent Educ.

[10] Goldszmidt M, Minda JP, Devantier S, Skye AL, Woods NN (2012). Expanding the basic sciences debate: The role of physics knowledge in interpreting clinical findings. Adv Health Sci Educ.

[11] Schmidt HG, Norman GR, Boshuizen HPA (1990). A cognitive perspective on medical expertise: Theory and implications. Acad Med.

[12] Norman GR, Trott AD, Brooks LR, Smith EKM (1994). Cognitive differences in clinical reasoning related to postgraduate training. Teach Learn Med.

[13] Williams RG, Klamen DL (2012). Examining the diagnostic justification abilities of fourth-year medical students. Acad Med.

[14] Cianciolo AT, Williams RG, Klamen DL, Roberts NK (2013). Biomedical knowledge, clinical cognition and diagnostic justification: A structural equation model. Med Educ.

[15] Alberstone CD, Benzel EC, Najm IM, Steinmetz MP (2009). Anatomical Basis of Neurologic Diagnosis.

[16] Moore KL, Dalley AF, Agur AMR (2013). Clinically Oriented Anatomy.

[17] Gilhooly KJ, McGeorge P, Hunter J (1997). Biomedical knowledge in diagnostic thinking: the case of electrocardiogram (ECG) interpretation. Eur J Cogn Psychol.

[18] Woods NN, Howey EH, Brooks LR, Norman GR (2006). Speed kills? Speed, accuracy, encapsulations and causal understanding. Med Educ.

[19] Norman G, Young M, Brooks L (2007). Non-analytical models of clinical reasoning: the role of experience. Med Educ.

[20] Finnerty EP, Chauvin S, Bonaminio G, Andrews M, Carroll RG, Pangaro LN (2010). Flexner revisited: The role and value of the basic sciences in medical education. Acad Med.

[21] Irby DM, Cooke M, O’Brien BC (2010). Calls for reform of medical education by the Carnegie Foundation for the Advancement of Teaching: 1910 and 2010. Acad Med.

[22] Ling Y, Swanson DB, Holtzman K, Bucak SD (2008). Retention of basic science information by senior medical students. Acad Med.

[23] Brooks WS, Panizzi Woodley KTC, Jackson JR, Hoesley CJ (2015). Integration of gross anatomy in an organ system-based medical curriculum: Strategies and challenges. Anat Sci Educ.

